# Steroid Assays in Paediatric Endocrinology

**DOI:** 10.4274/jcrpe.v2i1.1

**Published:** 2010-12-08

**Authors:** John W. Honour

**Affiliations:** 1 University College London Hospitals, London, England; +44 771 090 1800john.honour@uclh.nhs.ukClinical Biochemistry, University College London Hospitals, London, W1T 4EU, England

**Keywords:** Steroid, adrenal, adrenal tests assay

## Abstract

Most steroid disorders of the adrenal cortex come to clinical attention in childhood and in order to investigate these problems, there are many challenges to the laboratory which need to be appreciated to a certain extent by clinicians. The analysis of sex steroids in biological fluids from neonates, over adrenarche and puberty present challenges of specificities and concentrations often in small sample sizes. Different reference ranges are also needed for interpretations. For around 40 years, quantitative assays for the steroids and their regulatory peptide hormones have been possible using immunoassay techniques. Problems are recognised and this review aims to summarise the benefits and failings of immunoassays and introduce where tandem mass spectrometry is anticipated to meet the clinical needs for steroid analysis in paediatric endocrine investigations. It is important to keep a dialogue between clinicians and the laboratory, especially when any laboratory result does not make sense in the clinical investigation.

**Conflict of interest:**None declared.

## INTRODUCTION

The analysis of steroids for investigations in paediatric endocrinology is a challenge for the laboratory. There are many developmental events and pathological disorders to be considered in providing such a service. The laboratory must also be on the look out for anomalous results as well as keep abreast of new analytical developments that may improve the service. The specificity of a steroid assay for one analyte is often not assured within a patient sample. In practice, a result cannot be transferred with the patient if investigations move from one centre to another because of assay variabilities. These factors are attributed to interferences and inaccuracies probably through the differences in the antibodies used in the assays, also lack of standardisation of reagents and standards. These are now issues being addressed particularly for testosterone (1, 2, 3, 4). Certified reference materials in USA and Europe (native samples with defined hormone concentrations) have been available for many years as targets for hormone accuracy, but have not been widely used. Immunoassays for peptide hormones are prone to interferences from auto-antibodies and antibodies that react with reagents (heterophilic antibodies-mouse antibodies in patient samples that react with monoclonal reagent antibodies). In some (sandwich) assays, mainly for peptide hormones, false low results are due to high-dose hook effects. These issues will not be covered in the present review but have been addressed recently (5). Paper chromatography, thin layer chromatography (TLC) on silica and alumina, gas chromatography (GC) and high performance liquid chromatography (HPLC) have all been applied to the analysis of steroids in extracts of biological fluids, but only GC and HPLC are in routine hospital use and will thus be covered in some detail here.

Reference methods for certain hormones have been available in highly specialised centres. For steroids, those reference methods have relied upon stable-isotope dilution analyses with GC coupled with mass spectrometry (MS) (6, 7, 8). The dual selectivity of the separation of a mixture of components and specific ion detection enables exceptionally high accuracy without interferences (9, 10, 11, 12, 13). Some immunoassays have approached that level of accuracy, but only when the method includes extraction and chromatographic purification, again limited to very specialised laboratories (14, 15). A number of novel mass spectrometric techniques (fast atom bombardment, for example) looked to have the potential for more general analytical use (16), but did not stand up to rigorous clinical use and needed highly skilled operators. Tandem MS now looks to truly have the potential to improve the quality of assays needed in clinical practice. 

Reference intervals are essential for interpretation of test results. The derivation of these ranges is difficult and expensive and complicated in normal children by issues of consent and insufficient numbers for statistical analysis. Samples from the outpatient paediatric population and hospitalised patients without endocrine disease (surgical patients) are numerous and are a useful mechanism to overcome supply of reference materials (17, 18).

## BACKGROUND

**Technology**

Colorimetric assays for steroids were the first chemical means for the quantitative analysis of steroids, replacing bio-assays that had little use in paediatrics because of low hormone concentrations and the large sample volumes needed to get a measurable effect. Porter-Silber chromogens are formed when the dihydroxyacetone side-chain of 17-hydroxycorticosteroids (cortisol) react with phenylhydrazine. The Zimmerman reaction involves reaction of 17-ketosteroids (androgens) with dinitrobenzene. These tests also were not specific enough for clinical paediatric use and have largely become obsolete apart from a few countries, mostly outside Europe. In routine clinical practice, immunoassays have provided much valuable information and will continue to be used. Radioimmunoassays have been largely replaced with alternative labels. There are many problems with assay specificity. In any automated method in the laboratory, where parts of the equipment perform repeated steps in the process, there can be problems with sample carry-over when a sample containing high concentrations of the analyte precedes a sample with low concentration. Mass spectrometric techniques have much promise and there will be changes in endocrine laboratory services. New reference ranges will be needed and this will be a challenge for paediatric laboratories. Bioassays have been introduced using cell preparations, which enable more reproducible results than the classic experiments to assess biological activity of extracts when tested, for example, in adrenalectomised rats or immature animal. These novel assays are not yet in use routinely, but have a place in some investigations that will be considered later in this review.

**Figure 1:**
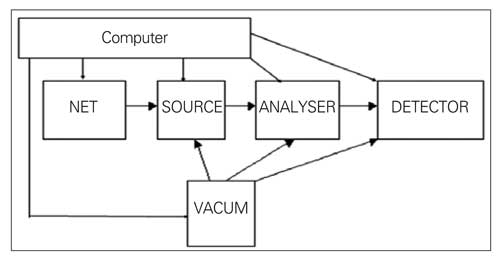
Schematic layout of a mass spectrometer. In tandem mass spectrometry (MS) the analyser would in principle comprise three analysers (quadrupoles), the middle unit being a collision cell. An ion focussed in the first MS would be fragmented in the collision cell. In the third MS daughter ions would then be focussed to the detector

MS is widely used in clinical chemistry and the applications can be appreciated with some understanding of the mechanics of the component parts. The analytes can be drugs, steroids, amino acids and organic acids as small molecules. Newborn screening is an area, where MS has brought major advances (19). There are some basic features of MS and a number of terms will be encountered when reading about the technology. Chromatographic separation is often coupled with MS detection and this is called a hyphenated method. The components of the sample after separation in gas or liquid chromatography are introduced into the vacuum of the mass spectrometer. MS is a technique in analytical chemistry based on measuring chemicals as ions in the gaseous state according to their weights (strictly speaking, according to mass and charge ratio [m/z]). MS analyses charged particles. A mass spectrometer can be divided into a sample inlet, source, analyser and detector, all controlled by a computer (Figure 1). The mass spectrometer produces ions in the source from molecules in a gas or handles ions delivered in a gas or spray to the MS. In the ionisation process, fragment ions may also be produced. All mass spectrometers operate at high vacuum to minimise ion-molecule reactions, scattering and neutralisation of ions. After ions are formed in the source, they are accelerated into the mass analyser. Once the ions enter the analyser, they are usually sorted in an electrical field or on the time to pass to the detector (time of flight analyzer). The quadrupole mass spectrometer is probably among the commonest instrument designs now being used in clinical work, although ion traps have high sensitivity. The quadrupole analyser consists of four rods. Rods operate in opposite pairs and each carries an Rf voltage. Only ions of the proper m/z value can successfully traverse the entire filter to reach the detector. Other ions are drawn into the rods. When scanning over a mass range, the DC potential is varied linearly with the Rf/DC ratio held constant and an entire spectrum is obtained. 

The mass analyser separates ions, either in space or in time, according to their mass to charge ratios. After the ions are separated, they are detected and data transferred to the computer for analysis. Most mass spectrometers use a photomultiplier detector tube. Data analysis includes plotting spectra, background subtraction facility, library search and chemical formula development, processing of quantitative data. Several spectra can be taken per second. Quadrupoles cover a wide mass range (typically up to 1,000 Daltons) that is in essence extendable when looking at multiply charged particles that have mass to charge ratios within a lower range than the molecular weights. Many compounds will have the same nominal or integral mass and will not be distinguished by the usual low resolution mass spectrometers. Resolution is defined according to the mass of the molecules separated by a valley, and is a function of mass and the difference between the 2 accurate masses. 

A mass spectrum shows the intensities of the range of ions (m/z 50 to 900 is a typical range) from an analyte and is a qualitative fingerprint of the molecule. Accurate quantitative analysis is possible with MS and some regard the technique as a reference method, though this may not always be the case. The acquisition of an MS can be justified by many laboratories through cost savings largely from lower reagent costs. In order for mass spectrometry to be effective, within the organisation of a clinical laboratory, a minimal analysis time and effort in sample preparation was a key element. 

The accuracies of steroid methods are variable and results sometimes not clinically sound for all of these components, particularly for low concentrations of certain steroids in children (20, 21, 22, 23, 24, 25). There is now a need to move to accurate methodology fit for clinical purpose. There will be a need for change in laboratory practices and external quailty assurance (EQA) must move away from the traditional approach of matching results with a peer group and ignoring the differences between methods (1, 26). Laboratories have to consider other factors such as sample carry-over in automated methods (27). There is a need for systematic reviews of tests in relation to diagnostic need (28) this has been started for steroid assays (29).

**Samples**

Blood, saliva and urine are fluids available for steroid analysis. It is important to consider the information that is available from different tests. Each method requires reference range appropriate to the development of the child and related diagnostic significance (30). Steroids in blood are largely bound to proteins (albumin and specific proteins such as sex hormone-binding globulin (SHBG)). The free steroid in blood is filtered at the salivary gland and kidney, and steroid concentrations in saliva and urine correlate with free hormone in blood. In the investigations of thyroid function, there are many methods that quantify free hormone concentrations in plasma. That has not been widely used in the steroid field. Assays for steroids after equilibrium dialysis or ultrafiltration have been described for cortisol (31). Free testosterone can be determined by analogue methods (32), ammonium sulphate precipitation (33), dialysis and ultrafiltration. Five routine assays have been compared with a GC-MS method based on ultrafiltration (34). The methods are not amenable to automation and were not considered important in an Endocrine Society review of testosterone determinations (1, 35). Indices of free hormone are used by some, based on a percentage of the total steroid that can be bound to a concentration of the specific binding protein, i.e. free androgen index

FAI=(serum testosterone/SHBG concentration)X100

Two assays are needed, both of which are subject to error, particularly in prepubertal boys, when testosterone is <5 nmol/L and SHBG above 120 nmol/L.

Blood samples can have large changes in steroid concentrations over a 24^h^ period and timing can be critical, so the laboratory need to be informed of time of collection of any sample to assist interpretation of results. Plasma cortisol can be 200-800 nmol/L around 0800h and less than 150 nmol/L at midnight. This is often depicted as a sine wave curve to illustrate a diurnal rhythm. In fact, there is a strong pulsatile pattern during the night with 3 or 4 peaks of increasing intensity starting around 0200^h^ to 0300^h^ with a final peak between 0600^h^ and 1000^h^ (36). Antenatal dexamethasone administration in pregnancies at risk of preterm delivery is an established procedure to decrease neonatal mortality. This can affect the hypothalamic-pituitary-adrenal axis in the neonate and influence hormone tests (37). Whoever collects samples should check laboratory guidelines for sample size and container, because material in the containers and separating agents and preservatives can affect assay performances (38). It may not always be possible to get a sample sufficient for all the tests wanted, so a priority should be discussed with the laboratory to optimise use of sample. Laboratories need to preserve samples in the state they left the body, so separation of plasma from blood cells in a timely manner and suitable storage around freezing temperatures or below need to be observed and monitored (39, 40, 41).

Saliva samples have some attraction for paediatric investigations, because collection is less invasive (42, 43 and 44 for reviews). There are a number of techniques to improve saliva flow (45). Like urine, saliva steroids reflect the free hormones in the blood. The steroids concentrations are thus normally very low (cortisol around 5 nmol/L) and sensitive assays are needed. 11-Hydroxysteroid dehydrogenase activity (HSD11B1) is active in salivary glands, so saliva has high concentrations of cortisone and a cortisol assay must be used that does not detect cortisone as well (46). The loss of diurnal rhythm of cortisol can be detected in screening for Cushing’s syndrome (47, 48, 49). Saliva samples can be taken for analysis of 17-hydroxyprogesterone (17-OHP) or androstenedione (AS) to monitor congenital adrenal hyperplasia (CAH) treatment (50, 51). Because of the variations in steroid production and low concentrations of steroids in blood and saliva, urine has been a useful biological fluid for analysis. 

Free cortisol excretion rate in urine can be determined. This is an important index of cortisol excess, but should never be used to demonstrate adrenal suppression. Immunoassays for cortisol in urine are not specific, being subject to cross reaction from cortisone (52) and steroid metabolites (53, 54). Steroids are inactivated in the liver through a number of reductive enzyme catalysed reactions and metabolites are conjugated with glucuronic and sulphuric acid to aid urinary excretion. In the neonate and for the first six months of life, there are a number of active enzymes (hydoxylases at carbons 1, 6, 15, 18), which adds to the number of steroids requiring identification in normal and pathological states. From a 24h collection, the combined excretion rates of corticosteroid metabolites come close to production rates. In young children, a 24h collection is difficult to obtain reliably, for much diagnostic work analysis of steroids in a spot sample gives useful information. Some laboratories are able to accept disposable diapers (free of any faecal soiling) for certain analyses (55). Urine can be obtained by squeezing, pressing (hydraulic or wine press) or centrifugation (fruit juice extractor). 

In paediatrics, there are wide changes in reference ranges for age, gender and stage of development that influence the mix of steroids present in the sample. There are problems with samples from preterm babies and neonates (interferences from adrenal fetal zone steroids), adrenarche (increase in adrenal androgen secretion) and puberty (changes in concentrations of binding proteins, as well as pituitary and gonadal hormones). Furthermore, when providing assays for the range of clinical conditions, then results are encountered that fall outside the range of assay calibration. Samples then need to be diluted. Ideally, the matrix needs to be preserved, so use of the assay zero standard is the best way to achieve this without altering the dynamics of the assay. Many publications attest to the difficulties created clinically from misinterpretation of steroid results (see # 56 for 2 examples around testosterone assays).

## IMMUNOASSAYS OF STEROIDS

In the 1960’s, immunoassay revolutionised the clinical laboratory services for hormones (57). The low concentrations in children of certain steroids often meant larger samples were needed to achieve sensitivity in the assays. An extraction and concentration step was needed in some cases (58). In order to reduce total sample size for paediatric samples, especially when several tests were needed, some laboratories developed chromatographic separation of a single sample extract before immunoassays of the separate components-an extremely demanding and time-consuming approach (14, 15, 59, 60, 61, 62, 63, 64). Some automation of the procedure is possible (65). Specificity of steroid assays is also improved by chromatography before immunoassay (for example paper chromatography (66) or column chromatography with celite (67, 68, 69) Kieselguhr (70) or using Sephadex LH-20 (71) or Lipidex, a lipophilic derivative of Sephadex (72, 73). Since steroids are largely bound to proteins, it is usual to measure the concentration of the total hormone in plasma after displacement of the steroid by extreme pH, heating or addition of a synthetic steroid. 

**Figure 2:**
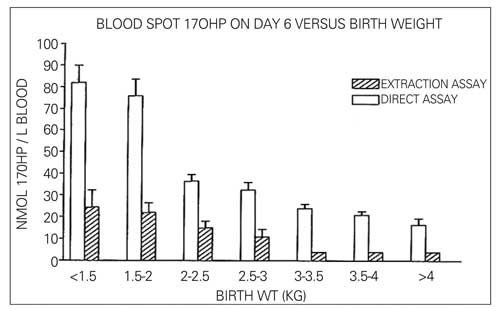
Direct assays for 17-hydroxyprogesterone (17-OHP) give higher results than when a solvent extract of a sample is analysed. Interferences in the assay from fetal adrenal steroid sulphates are removed by the extraction from reference 75, with permission

Immunoassays have evolved over the years (74). Initially, assays were set up in-house using reagents that were generated by the laboratory, or obtained from other laboratories or purchased from commercial sources. The key components were a standard to generate a graph of signals from the range of hormone concentrations (calibration curve), an antibody and a label. Antibodies were initially created by injection of mice, rabbits or sheep with a hapten. For steroids, it is necessary to couple the steroid to a protein, such as bovine serum albumin of limpet haemocyanin, to create a significant hapten. Specificity was improved by affecting the union of steroid to protein at a position in the steroid remote from the functional groups. After several injections of the hapten, high antibody titres were generated and serum from the animals could be diluted to give large volumes of the assay-ready antibody preparation. As specificity of the antibody recognition of a steroid improved, then confidence was gained to use biological samples directly in the assay tube. Prior to that time, steroids were extracted into an organic solvent that was dried and reconstituted in assay buffer for the analysis. The extraction step was certainly important to separate for neonatal samples, in which the presence of fetal adrenal zone steroid sulphates needed to be stripped from free steroids in the samples, so as to reduce to interference in the immunoassay (see Figure 2 from reference 75 for an illustration of the lowering of 17-OHP results in an assay based on solvent extracts compared with plasma itself). In some cases, steroids in the organic extract were purified by chromatography (76) and the eluates from an HPLC could be collected in timed fractions (77). 

At the outset, carbon-14 and tritium labelled steroids were commonly used and liquid scintillation counting was required for detection of beta-radiation. The label was usually present in the assay tube at a concentration to react with around 50% of the binding sites on the antibodies. Addition of steroid in the sample thus reduced the amount of bound label (competitive immunoassay). After the reaction of analyte and label with the antibody, the free and antibody fractions had to be distinguished. The free fraction could be absorbed from the sample with charcoal that could then be precipitated with the aid of centrifugation. Waste disposal was a big problem, so gamma ray emitting isotopes (iodine 125, for example) were attractive, although they had short half lives, so reagent had to be renewed on a regular basis. To introduce iodine, labelling of a steroid usually requires the use of a derivative of the steroid that will react with iodine. Tyrosine and histamine conjugates of steroids have been common substituents for this purpose, but this then makes the label structurally different from the natural steroid in a biological sample, so the compounds react differently with the antisera (78). Multi-well gamma counters helped with rapid batch processing of 10 or 16 samples at a time. The antibody-bound fraction could be precipitated after the addition of a second antibody (donkey antisera to the mouse, rabbit or sheep protein), that increased the molecular size of the complex. Later techniques were developed to bind the antibody to the wall of a reaction tube (coated tube) or to particles (beads or magnetic particles); the free fraction in solution could then be decanted at the end of the reaction. 

Commercial kits for 17-OHP typically are designed to detect elevated concentrations in heterozygotes for classical disease and patients with non-classic forms-a worthwhile test in the investigation of patients with androgen excess and infertility. Kits, however, to detect the classical form of the disease would not be economically viable. Interferences in 17-OHP assays can be encountered in the newborn period often from the fetal adrenal zone steroids (DHAS, pregenenolone) (79). In patients with CAH due to 21 and 11-hydroxylase deficiency, the high concentrations of cortisol precursors can lead to falsely elevated results for cortisol (80, 81).

Monoclonal antibodies were successfully generated for many analytes, but for steroids they did not initially prove to be as good as the polyclonal animal raised antibodies. Now, monoclonal antibodies are attractive, because the supply of a characterised antibody can be sustained (82, 83, 84). Some changes in hapten have assisted antibody quality (85). A few methods are based around anti-idiotype assays (86, 87). Since mice are used for monoclonal experiments, samples from patients exposed to mice (or treated with mouse monoclonal antibody therapies) can introduce heterophilic antibody reactions (88). Unusually low results for steroids can be found (89) and appropriate steps need to be taken to counteract the heterophilic antibodies (90, 91). To overcome concerns over safety and stability of reagents, alternative labels were introduced (chemiluminescent, fluorescent, as examples) (92), and from that time automation gradually took over (reviewed by Wheeler # 93). With the introduction of multi-channel automated analysers, it became possible for the laboratory to analyse all the components for results of a panel of hormones (cortisol, progesterone, oestradiol, testosterone, T3, free T4, DHAS, LH, FSH, TSH, prolactin, ACTH) in one sample over a few hours. Multi-channel, automated methods really needed to have the same incubation steps for each analyte and inevitably optimisation for certain assays had to besacrificed. In general, steroid assays take longer times to reach equilibrium than peptide assays.

Automated immunoassay systems have been evaluated in the paediatric setting and found to be acceptable (94, 95, 96). Problems have inevitably been encountered with automated methods in the laboratory. In immunoassays, results for testosterone in newborns have been reported to be grossly elevated (20) due to interferences from fetal and placental steroids. One publication reported that, when samples were tested for testosterone after the same sample had just been tested for oestradiol (97), high concentrations of testosterone were indicated. This was explained by carry-over of reagents in the sample probe, rather than sample, because direct steroid assays need to incorporate a means to displace steroid from binding proteins (for example, testosterone in the oestradiol assay). Such problems are not easy to resolve, because the formulation of assays is not described in detail by the instrument manufacturers. The information on cross reactivity is another area where kit information may not be entirely valid. Ideally, a displacement curve should be plotted for any potential interfering compound. Cross reactivity is then expressed from the 50% binding concentration of the interferent relative to the concentration of the analyte at that point in the calibration curve (then usually expressed as a percentage cross-reaction). Many kit manufacturers take an approach of adding a concentration of the interferent that is assumed to be greater than ever encountered in biological samples and assessing the concentration measured as a percentage of the amount added. DHAS can cross react in the testosterone assay on the Abbott Architect (98). 

A metabolite of an interferent can be more potent in the immunological reaction and create a falsely high result. Metabolites are not always available and indirect evidence is needed to support proof. For instance, in an androstenedione assay, elevated concentrations can be apparent in samples collected from patients taking spironolactone. The cross reactivity was reported to be less than 0.01%, but androstenedione returned to the normal range when treatment was withdrawn, suggesting interference, probably from the metabolites rather than the drug itself (Honour and Dawnay, unpublished) (see Table 1). Low concentrations of steroids are difficult to assay. Direct immunoassays are not sufficiently accurate and specific for oestradiol in children (23) or androgens in the investigation of disorders of sexual development (56, 99, 100).

**Table 1:**
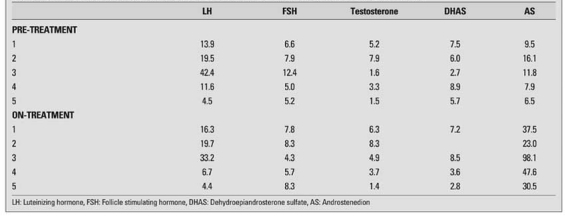
Endocrine results in patient with polycystic ovary syndrome before and on treatment with spironolactone

## GAS CHROMATOGRAPHY

Although many investigations have relied upon analysis of steroids in blood samples, the analysis of urinary steroids by GC has proved to be a very powerful tool with the ability to recognise most of the steroid disorders in children from characteristic steroid profiles (metabolomes) (101, 102). In fact, many of the recently discovered disorders of the adrenal cortex can be credited to this technique pioneered in 1970 by Cedric Shackelton, who has ever since made seminal contributions to this diagnostic area (see Table 2 for summary of these achievements). Steroids need to be extracted from urine; solid phase extraction is commonly used for this. Steroid conjugates are hydrolysed using glucuronidase and sulphatase enzyme preparation. After re-extraction of steroids, methyloxime-trimethylsilyl ether derivatives are formed. These make the steroids stable for high temperature GC separation and direct fragmentation in the MS. Steroids eluting from a GC column can be detected non-selectively by a flame ionisation detector, the identification is by retention time. If the effluent is directed to a mass spectrometer, then hundreds of spectra can be taken from mass to charge 50 to 1000 over a 30 minute GC run. Each spectrum is a fingerprint of the steroid. For the detection of gross changes in steroid production, a spot sample of urine can provide enough information in a steroid profile from whether individual and groups of steroids are low, normal or raised. In CAH, there will be low levels of cortisol metabolites, raised concentrations of intermediates (such as 17-OHP metabolites), and low or raised androgens, depending on the affected enzyme. As a result of the pattern of high cortisol production during the night, the highest urinary excretion rates of the cortisol metabolites is between 1000h and 1800h (103), so it is not wise to collect spot samples in the early morning or afternoon for assessment of cortisol production on this basis. Ratios of metabolites are diagnostic of 5-alpha reductase deficiency and defects of HSD11B1 (high cortisone) and HSD11B2 (high cortisol). 

In order for steroids in plasma to be detected in GC methods, it was necessary to make derivatives that were suitable for sensitive electron capture detection. This was a difficult detector to keep for routine use and will not be considered here. GC-MS has been found also to be a very reliable quantitative method for steroids in plasma such as DHAS, 17-OHP, AS, testosterone, 17-hydroxpregnenolone, 11-deoxycortisol and DHAS (7, 8, 9, 10, 11, 12, 13). It is superior to other methods, because of the improved specificity of assays through separation of steroids in the sample extract, which is needed particularly for tests on newborn infants. A deuterium labelled internal standard steroid of high purity is used for what is effectively an isotope dilution analysis. The internal standard is added at the start of the assay, so acts as a recovery marker for the extraction and chromatography. Heptafluorobutyrate derivatives are most used, because of their ease of preparation and minimal fragmentation in the mass spectrometer. After GC separation, the steroids are fragmented usually by electron impact, and ions for the analyte and the internal standard are monitored by the MS. Specificity in the assay can be demonstrated from the relative responses for a quantifier and a qualifier ion for each compound. Calibration curves are linear for low concentrations through normal levels to grossly pathological levels in patients with CAH, where levels up to 1000 nmol/L can be seen. There is 97-103% recovery of steroid added to samples across a wide range of concentrations. Comparison of results with a number of clinical samples assayed by a direct and an extracted RIA showed close agreement with extracted RIA’s. Most laboratories will not measure 17-OHP in the first 3 days after birth because of false high results due to interference from fetal adrenal zone steroids like 17-hydroxypregnenolone and its sulphate (79). The GC-MS method is not subject to these interferences and reliable results can be obtained in affected children within 12h of birth. Internal QC shows low CV’s compared with immunoassay methods. In external EQA, the GC-MS method performances have also been impressive with CV’s less than 3%. Only 500 microlitres of sample is normally used but when necessary 50 microlitre sample can be used without compromising a result. Normal ranges at different ages and genders are available, as well as reference ranges for Synacthen tests. These values are different to results from immunoassays. This assay has been compared with MS/MS, but the GC/MS has a very high standard to beat. Any child with ambiguous genitalia at birth needs a 17-OHP result as soon as possible; this method meets the criteria, with results less than 3 nmol/L in normal infants from the first day of life, and clearly elevated above 100 nmol/L in a child with 21-hydroxylase deficiency. 

Just as profiles of steroids by GC and GC-MS reveal information to recognize abnormal patterns with adrenal disorders then GC-MS-MS provides additional information. GC-MS–MS in product ion scan mode enables the recognition of most steroids in the sample through the combination of retention time and fingerprint of the mass spectrum. The nature of unknowns can also be approximated (104). GC coupled with tandem mass spectrometry (GC-MS-MS) (MS1 selecting ions, MS2 scanning fragments) can give even greater power to identification of steroids. Recently, more than 120 steroids were characterised in the urine of patients with CAH due to 21-hydroxlase deficiency (104). The sum of glucocorticoid metabolites in urine (TCM) collected for 24^h^ approximates the daily production rate of (105, 106) and is an excellent test for adrenal suppression (107). The improved sensitivity of LC-MS-MS methods shown for androgens and oestrogens (to<10 pmol.L) will now allow earlier and more accurate assessments of pubertal development (108). Even before signs of puberty, girls had higher androgen and oestrogen levels than boys.

**Table 2:**
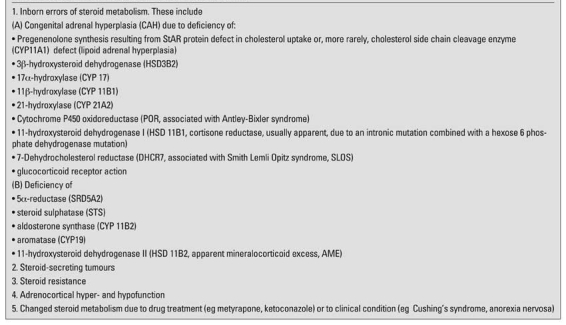
Applications of urinary steroid profile analysis by GC-MS

## LIQUID CHROMATOGRAPHY

Steroids can be separated by reverse phase HPLC and detected with UV spectroscopy for some diagnostic work (76, 109, 110, 111, 112, 113, 114, 115). In reverse phase chromatography, a typical elution order will be aldosterone, cortisol, DHAS, corticosterone, 11-deoxycortisol, AS, testosterone, DHA, 17-OHP and progesterone. This approach is only successful, when sample concentrations of the steroids are in excess of 200 nmol/L-i.e. for cortisol and DHAS under normal circumstances and raised concentrations of intermediates in patients with disorders of cortisol synthesis. Sample processing is minimal and results can be available within hours of sample receipt in the laboratory. 

In LC-MS, the effluent from HPLC is directed through a capillary to the source of the mass spectrometer. The temperature of the capillary is adjusted to a level, where the solvent is partially vaporized (Thermospray). This generates a supersonic jet of vapour that contains a mist of electrically charged particles. The ion chamber is evacuated and solvent continues to leave the droplets concentrating charge at the surface. Eventually, ions are expelled and leave the thermospray source through a small orifice in the sampling cone. Ammonium acetate is often incorporated into LC solvent systems to accelerate the ionisation process here. 

Cortisol, being in blood at 200 to 800 nmol/L as opposed to 17-OHP around 5 nmol/L, can be measured by LC/MS without the lengthy sample work-up and derivative formation needed in a GC-MS method. This methodology has also been used to establish the production rate of cortisol. Previously, radioactive methods had been used giving results around 18 milligrams per day (116). Clinicians were comfortable with that, because replacement treatment is usually at around that level, but no treatment regimen yet replaces the normal pattern of cortisol concentrations in blood over the 24 hours. When stable isotope dilution methods were used, cortisol production rate is 6 to 9 milligrams per metre squared with most secretion between 0200 and 0800h (117) as expected from blood concentrations when samples are taken at 10 minute intervals. Stable isotope dilution LC-MS has been used for 17-OHP in plasma (118).

Fast atom bombardment in the mass spectrometer was a technique of great promise some years ago, but did not get widely adopted and is rarely used today. A steroid extract could be dissolved in glycerol and put on the whiskers of a probe where the sample was desorbed and ionised by being bombarded with fast Xenon or caesium ions. Steroid conjugates did not need to be hydrolysed and mass spectral patterns were obtained of individual steroid (119). Abnormal results were seen for glucuronide conjugated steroids in CAH (16). When pregnancy urine was analysed by FAB MS, the profile from a normal pregnancy could be distinguished from a pregnancy with placental sulphatase deficiency by the presence in the latter of DHAS metabolites. The instrumentation needed regular cleaning and maintenance and was not suitable for processing large numbers of samples.

Liquid chromatography coupled with tandem mass spectrometry (LC-MS-MS) now has the potential for steroid analysis in paediatric samples. Tandem MS is a combination of mass spectrometers to improve analytical performance. More detail about the technology can be found in recent reviews (120). MS coupled with MS (tandem MS) enables sequential filtration, fragmentation and focussing of ions. The collision cell is similar to the quadrupole analysers at MS1 and MS2, so some systems use the term triple quad for a tandem mass spectrometer. Each MS can select specific ions or scan a mass range looking at a spectrum of ions. For quantitative MS, multiple reaction monitoring (MRM) is used. For a description of other modes, see Chace 2009 # 19 for a review. In an MRM, the first MS (MS1) affords sample purification by selecting an ion of mass to charge for the required analyte. Between the two MS sectors, a collision cell can produce a region for fragmentation of the ion that penetrated the first MS. The second MS (MS2) is set to transmit a fragment ion; characteristic of the analyte can be operated as a traditional MS. So in summary, ions are sorted in the first MS, fragmented, then sorted to monitor intensity of ions in second MS. The combination after LC separation thus adds sensitivity and specificity. 

In the source, neutral molecules are ionised, then accelerated into the mass analyser. In electrospray, ionisation takes place at atmospheric pressure. A large electrical potential is applied to the metal inlet needle bringing the sample out of the HPLC. As the HPLC effluent liquid leaves the nozzle, the electric field induces a net charge on the small droplets. As the solvent evaporates, the droplet shrinks and the charge density at the surface of the droplet increases. After further evaporation, the droplet explodes and release charged analyte ions. Most androgens and intermediate steroids are measured as positively charged ions; some corticosteroids are measured as negative ions, aldosterone, for example (121). In a few cases, derivatives are formed to assist ionization and assay sensitivity, notably for oestrogens (122,123) and steroid conjugates (124).

Stable isotopes are very useful in a number of ways in clinical investigations and make for ideal internal standards in quantitative MS analysis. A range of stable-isotope labelled steroids are commercially available (Table 3). In the LC analysis, the isotopic steroids move slightly faster than the unlabelled analyte; when 5 or more deuterium atoms are included, the two steroids will almost separate, so in effect, the carrier effects of the 2 that protect against system losses can be negated. Due to the natural distributions of isotopes of the elements, all compounds in a mass spectrometer give a cluster of ions, notably at M+1 and M+2 Daltons. If the stable isotope steroids had 2 deuterium atoms, then it effectively is indistinguishable from natural isotopes of the analyte. Ideally, a labelled steroid for use, as an internal standard in MS, should have a mass increment of 3-6 Daltons. Alternatives are isomers and analogues of the analyte that separate from the analyte. High sensitivity methods can be established, because only 2 or 4 ions need to be monitored for an effective analysis with the MS used in ion monitoring mode. The intensities of at least 2 ions from the analyte and 2 ions from the internal standard are recorded with time. A typical set of ions monitored is shown in Table 4. Sensitivity is increased about 500-fold compared with scanning mass range and processing ion signals relevant to the analysis. Response ratios for the ions are as seen in the spectrum. Usually, the most intense ion in the spectrum is used for the quantitative analysis and as described in the GC-MS section, the other ion act as qualifier ion to demonstrate specificity (125, 126).

Steroids can be determined singly in samples or as a profile. This technique will only be described in detail sufficient for clinicians to appreciate what the methods can offer to paediatric endocrine investigations and review the applications to date. Minimal sample preparation is an important advantage, saving time and cost. Protein separation being the simplest first step. The improved specificity of the results leads to more accurate results and new reference ranges will be needed to interpret LC-MS-MS results. Most of the assays are currently at the in-house stage, like when immunoassays were first introduced and results need to be considered carefully. Any abnormal result should be discussed with the laboratory staff, so that problems can be addressed. Interferences can still be encountered and these need to be catalogued, so that the method can be modified to overcome the problem. The equipment manufacturers are exploring options to provide reagent kits for analytes and profiles, so introducing an important level of assay standardisation so desperately needed clinically.

Steroids and vitamin D are being analysed by tandem MS in an increasing number of centres. Assays of steroids in blood, urine and saliva have been reported. As yet there has not been published much by way of QC performance to see if MS/MS is superior to GC-MS where very good precision have been shown by some users as well as poor reproducibility in other tests. Although in principle reference standard methodology is being used (127), the results do not yet meet the requirement one might expected. Reasons for this include matrix affects, procedural differences, calibration differences. The  laboratory has an important role to play in the method validation, but there is an obligation for clinicians to alert the laboratory if problems are seen with results out of keeping with the diagnosis.

Despite the apparent simplicity of LC-MS-MS methods, the technique suffers from suppression and enhancement of ionization from compounds co-eluting from the LC into the source of the MS – this is called matrix effect. This can seriously affect the accuracy of the method and requires extensive validation and continuous checking of data to detect interferences. The use of a stable isotope internal standard in most cases, but not all (128, 129, 130, 131, 132), will compensate for this, because the chemical similarity with the analyte means both steroids are affected equally. A number of validation experiments are used, such as recovery of standard addition, post-column infusion of standard, and sample dilution (133, 134, 135, 136). Since the method looks at mass to charge of a compound and its fragments in the collision cell, then any isomeric steroid will have to be known not to interfere. The isomers will often be separated in the LC. Compounds with the same molecular weight as the analyte are isobaric and have potential for being detected like the analyte (137, 138) or internal standard (139). For testosterone, as an example, this required extensive testing (127). Many compounds and especially phospholipids (140, 141, 142), are known to interfere in these methods; drugs can also suppress and enhance the signal (143, 144). These issues have been reviewed (145, 146) and laboratories have the responsibility to consider carefully the matters raised, so will not be debated further in this more clinically orientated review. 

In the LC-MS-MS, one can really only look for compounds for which retention times and transitions are known and monitored. By changing MS conditions at intervals over an extended HPLC separation, several steroids can be determined from plasma in one run and there are published reviews in paediatric use (147, 148, 149, 150). Several applications of LC-MS-MS profiles cover investigations of CAH, apparent mineralocorticoid excess, aldosteronism, Cushing’s syndrome and adrenal insufficiency (151, 152, 153, 154, 155, 156, 157) and disorders of sexual development (158, 159, 160). Reference ranges for steroids in LC-MS-MS methods will be essential, some have been produced (155, 161, 162).

LC-MS-MS has also improved confirmation of newborn screening results (163, 164, 165). Steroids in urine have also been analysed by LC-MS-MS techniques as free steroids after hydrolysis (166, 167, 168) and as conjugates (169). High throughput can be achieved in a number of ways. HPLC with small particles (uPLC) and turbulent flow (170) has speeded up the separation stage; on-line solid phase extraction (171) and column switching (172) are in use. Whatever system is used, the laboratory needs to look out for signs of contamination and carry-over (173).

**Table 3:**
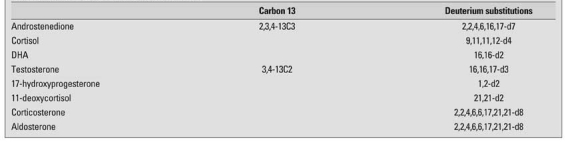
Examples of stable isotope labelled steroids

**Table 4:**
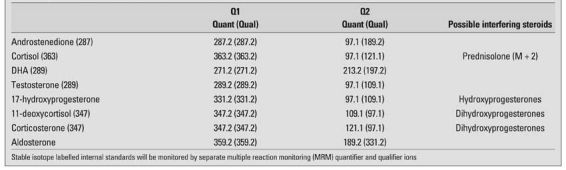
Typical transitions monitored in LC-MS-MS for steroids

## BIOASSAYS

Before chemical assays for hormones, the activity could be detected in vivo. Bioassays were insensitive and labour intensive. These tests included for androgens the growth of the capon comb and levator ani muscle, prostate and seminal vesicles of prepubertal rats injected with steroid extracts (174). A number of novel assays look at hormone activity (concentration) in vitro. Steroid activity using primary cells or cell lines that may have been modified genetically are in use. Three types of assay are based on receptor binding, cell proliferation and reporter genes.

A receptor binding assay is like a competitive steroid immunoassay with the antibody replaced by a receptor preparation. The cells may have an endogenous receptor (Cos-7 cells, for example) or cells modified to have a receptor inserted that expresses the receptor protein (MCF-7) (175). The simplest and most sensitive oestrogen assays look at proliferation of MCF-7 cells (E–screen) (176, 177). A-screen uses MCF-7 cells transfected with the AR (178). Reporter assays measure the level of expression of a steroid dependent reporter gene in response to steroid stimulation. Cell lines and yeast cell strains are transfected with the receptor gene and a reporter gene for an enzyme such as luciferase, beta-galactosidase or chloramphenicol acetyltransferase. When the cells are exposed to ligand, the reporter is activated. A substrate is added and signal detected by luminometry, spectrophotometry or fluorimetry (179). Such assays have been used for androgens in boys (180, 181, 182) and oestrogens in girls (183, 184, 185). These assays are not yet ready for routine use, but have provided useful information in research settings and in the search for endocrine disrupting chemicals in the environment.

## CONCLUSION

• Immunoassay has replaced chemical tests of steroids based on colorimetry and will remain important in the field of steroid testing. Some investigations of paediatric problems can be resolved with this technology but is subject to interferences. 

• Chromatography can be used before immunoassays to improve specificity, but is too labour intensive for routine use. 

• Gas-liquid chromatography with flame ionization and electrochemical detection and high performance LC with UV detection remains useful for measuring steroids at the higher ranges of concentrations in biological fluids (above 100 nmol/L). GC-MS of urinary steroids is an extremely powerful diagnostic tool for defects in adrenal cortical function and will remain important in revealing the nature of steroids in a sample without selection.

• MS is a powerful analytical tool for qualitative and quantitative analysis when coupled with gas and liquid chromatography. MS has been largely used clinically in specialist areas for analysis of steroids alone and in profiles. Tandem mass spectrometry (MS-MS) is becoming more attractive as a routine tool and will bring benefits of new technology to broaden applications.

• LC-MS-MS is often regarded as reference technology. Accuracy and specificity are better than immunoassay, but LC-MS-MS does not yet meet the higher precision standards achieved with GC-MS. 

• This review has considered the potential for tandem MS in steroid analysis. Comparisons of results with other methods and results of EQA suggest that LC-MS-MS often shows lower results, but immense performance variation probably through lack of skill, pressure to reduce analytical time, and calibration differences. Manufacturers are working to improve that by developing “kits”-a move that will be well received by clinical laboratories.

• Some progress has been made for peptides, but progress in this area is likely to be slow. Currently, proteins have to be digested to peptide fragments.

• Bioassays are unlikely to compete routinely with immunoassay or MS methods.

• Clinicians should always discuss unusual results with the laboratory, since there may be explanations and alternative investigations that can be analysed by a tandem mass spectrometric approach.
